# STTG-net: a Spatio-temporal network for human motion prediction based on transformer and graph convolution network

**DOI:** 10.1186/s42492-022-00112-5

**Published:** 2022-07-29

**Authors:** Lujing Chen, Rui Liu, Xin Yang, Dongsheng Zhou, Qiang Zhang, Xiaopeng Wei

**Affiliations:** 1 National and Local Joint Engineering Laboratory of Computer Aided Design, School of Software Engineering, Dalian University, Dalian, 116622 China; 2 School of Computer Science and Technology, Dalian University of Technology, Dalian, 116024 China

**Keywords:** Human motion prediction, Transformer, Gragh convolutional network

## Abstract

In recent years, human motion prediction has become an active research topic in computer vision. However, owing to the complexity and stochastic nature of human motion, it remains a challenging problem. In previous works, human motion prediction has always been treated as a typical inter-sequence problem, and most works have aimed to capture the temporal dependence between successive frames. However, although these approaches focused on the effects of the temporal dimension, they rarely considered the correlation between different joints in space. Thus, the spatio-temporal coupling of human joints is considered, to propose a novel spatio-temporal network based on a transformer and a gragh convolutional network (GCN) (STTG-Net). The temporal transformer is used to capture the global temporal dependencies, and the spatial GCN module is used to establish local spatial correlations between the joints for each frame. To overcome the problems of error accumulation and discontinuity in the motion prediction, a revision method based on fusion strategy is also proposed, in which the current prediction frame is fused with the previous frame. The experimental results show that the proposed prediction method has less prediction error and the prediction motion is smoother than previous prediction methods. The effectiveness of the proposed method is also demonstrated comparing it with the state-of-the-art method on the Human3.6 M dataset.

**Supplementary Information:**

The online version contains supplementary material available at 10.1186/s42492-022-00112-5.

## Introduction

Human motion prediction is the prediction of future poses based on a provided sequence of observed poses. It has promising applications in areas such as human-robot interaction, automatic driving, human tracking, and medical care. Nowadays, motion capture equipment can be used to accurately obtain human skeleton sequences. Therefore, it is feasible to use these sequences to predict the future poses of the human body. The human motion prediction problem is usually formulated as a sequence modeling problem, and a common approach to solving this problem is to model contextual information in the temporal dimension to capture the temporal dependence between successive frames.

In previous research, the majority of most methods have used sequential autoregressive or sequence-to-sequence encoder-decoder models. However, as human motion is a stochastic process, the capture of long-term historical information is difficult, so it is easier to generate static poses with an increasing prediction range. Therefore, motion prediction should depend on not only the temporal relationship between sequences, but also the spatial coupling relationship of different joints in motion. For example, in the action of ‘walking,’ the two arms should swing in opposite directions, so that the joints of the two arms influence each other during the process of ‘walking.’ Spatio-temporal dependencies have also been considered in action recognition research [1, 2], which further improve the recognition rate of actions. Recently, there has also been research that takes the spatial dependency into account. Li et al. [3] captured spatial dependencies through convolutional filters, but the dependencies were heavily influenced by the convolutional kernel. In addition, Mao et al. [4] used graph neural networks to simulate spatial correlation.

Past research indicates that relatively complex networks have generally been required to consider the temporal and spatial dependencies simultaneously, In addition, transformer models have become increasingly popular in computer vision fields and achieved unexpected performance in recent years. Compared with other neural networks, a transformer is completely based on attention mechanisms, there is no complex network structure and the number of parameters is small. Even the most primitive transformer structure may produce comparable results to a complex neural network. Therefore, through previous research, the transformer was introduced as a replacement for previously-used temporal modeling and combined transformers with other neural networks to model the spatio-temporal dependencies, thus effectively capturing more correlations in both temporal and spatial dimensions.

Affected by the cumulative error, the prediction error increases gradually with the prediction length. Moreover, when the prediction error increases suddenly, the “frame skipping” phenomenon occurs, and the predicted motion becomes stiff. Given the continuity of human motion, this paper presents a prediction revision module based on fusion strategy. The current prediction frame can be fused with the previous frame, to effectively reduce the prediction error and improve the continuity of the prediction action.

In short, the main contributions of this work can be summarized as follows.

- A spatio-temporal network STTG-Net consisting of a temporal transformer and spatial graph convolutional network (GCN) modules is designed. The temporal transformer can extract the global temporal correlation, and the spatial GCN can capture the local spatial coupling of the joints.
- A prediction revision module is proposed, which can effectively reduce the prediction error and improve the smoothness of the prediction sequence, thereby alleviating the problem of error accumulation.
- In the short-term motion prediction task, fewer parameters can be used, resulting in better prediction performance on the Human3.6 M dataset, and for non-periodic actions, the prediction effect is improved.

## Related work

The purpose of human motion prediction is to predict the trend of human motion based on observed human motion. As the frontier research direction of artificial intelligence, this technology has been widely followed and studied. The early traditional methods [5–11] were able to effectively model a single simple motion through mathematical calculations. With the development of deep learning and large-scale motion datasets, deep learning methods have become a better choice for human motion prediction compared to traditional methods. Since human motion prediction is a highly time dependent task and recurrent neural networks (RNNs) are well suitable for time series data, many works have applied RNN and their variants to solve this problem. In addition, some other works have attempted to take advantage convolutional neural networks (CNNs), generative adversarial networks (GANs), and more to solve this problem. Therefore, the related works are roughly divided between RNN-based methods and others.

### RNN based methods

Fragkiadaki et al. [12] constructed Encoder-Recurrent-Decoder and 3 LSTM layers, combined them with non-linear multilayer feedforward networks to predict motion trends of human skeleton in videos, and synthesized novel motions while avoiding drifting for long periods. To dynamically model the entire body and individual limbs, Jain et al. [13] proposed the S-RNN model, using a structural graph of nodes and edges composed of LSTMs for motion prediction, however, they ignored the problem of discontinuity between the observed and predicted poses. In addition Martinez et al. [14] solved the discontinuity problem by using a simple gated recurrent unit (GRU) with residual structure and demonstrated the effect of modeling one particular velocity. In order to synthesize complex motions and generate unconstrained motion sequences, Zhou et al. [15] proposed an auto-conditioned RNN model capable of generating motion sequences of arbitrary length and without the problem of stiffness. For static joints in prediction, Tang et al. [16] proposed a modified highway unit that effectively eliminated static poses by summarizing the historical poses associated with the current prediction based on RNN as well as the frame attention module. To guide the model to generate longer-term motion trajectories, Gopalakrishnan et al. [17] used derivative information as a computational feature in a neuro-temporal model with a two-level processing architecture containing a top-level and bottom-level RNN. The hierarchical motion recurrent network proposed by Liu et al. [18] used LSTM to model the global and local motion context hierarchically, and captured the correlation between joints by using Lie algebra to represent the skeleton frame. Corona et al. [19] proposed a context-aware human motion prediction method, which used a semantic graph model to build the influence by the spatial layout of the objects in the scene, and introduced an RNN to improve the accuracy of human motion prediction. In order to combine the influence of human trajectory on motion, Adeli et al. [20] used GRU to encode trajectory and pose information to solve the task of predicting both human trajectory motion and skeletal pose in an end-to-end structure. An RNN has excellent time modeling ability, but most works using RNN modeling ignored the spatial correlation between human joints.

### The other methods

Li et al. [3] considered both invariant and dynamical information of human motion and used a multilayer convolutional sequence-to-sequence model to learn features in space and time, resulting in more accurate predictions. Considering that the degree of activity of each part of the body during movement is different, Guo and Choi [21] divided the body structure into five non-overlapping parts based on the human body to learn the local structural representation separately and obtained better results in long-term prediction. Similarly, Li et al. [22] further improved the idea of Guo and Choi [21] to divide the human body into only five parts, constructing an encoder-decoder structure composed of multiscale graphs to extract human motion features at different scales and further improve the prediction performance. Barsoum et al. [23] tried to use a GAN to produce prediction output, and a Gaussian distribution vector z was added to GAN to increase the diversity of the predicted sequences. Two global complementary discriminators were introduced in the adversarial geometry-aware encoder-decoder framework proposed by Gui et al. [24] to improve the accuracy of long-time motion prediction through both local and global discriminators. In order to change the end-to-end training method of the human motion prediction task, Wang et al. [25] transformed it into a reinforcement learning problem by proposing a reinforcement learning formulation and an imitation learning algorithm that extended the generative adversarial imitation learning framework to be able to make accurate predictions of poses. Pavllo et al. [26] proposed a quaternion-based pose representation method, which solved the ambiguity and discontinuity caused by Euler angle and axis angle representation, and presented two versions using RNN and CNN, respectively. The structural training made predicted pose more accurate and the error smaller, but the conversion to four-dimensional space was relatively complex. Mao et al. [4] designed a simple feed-forward deep neural network, different from a pose space, which encoded temporal information in trajectory space via discrete cosine transform (DCT) based on the residual structure. The temporal variation of each human joint was represented as a linear combination of DCT coefficients, using a GCN to model the spatial dependence between joints. Building on this work, Mao et al. [27] later proposed a motion attention-based model to learn spatio-temporal dependence by forming motion estimates from historical information. Estimates were combined with the latest observed motion, and the combination was then fed into a GCN-based feedforward network. Recently, Mao et al. [28] investigated the use of different levels of attention, applying attention specifically to three different levels of the whole body, body parts and individual joints, and introduced a fusion module to combine this multi-level attention mechanism, achieving better performance. The advantages of GCNs were also found experimentally by Hermes et al. [29], who designed a spatio-temporal convolution with a GCN to extract spatio-temporal features, using an expanded causal convolution to model temporal information, which also contains local joint connectivity, to obtain a lightweight autoregressive model. In contrast, Martínez-González et al. [30] proposed a non-autoregressive transformer model to infer pose sequences in parallel, with self-attention and encoder-decoder attention, and added a skeleton-based activity classification to the encoder to improve motion prediction through action recognition.

A CNN generally abstracts dependencies between sequences by performing convolution operations in temporal dimension, but it is not as effective at learning sequence relationships over a longer period. A GCN can effectively learn temporal dependence of motion sequences through the supervised learning of generators and discriminators, but GANs are relatively difficult to train and their parameter tuning is complicated. Although RNNs are more suitable for processing data with temporal dependencies, their ability to learn long-time correlations remains weak, whereas transformer [31] can model global dependencies of inputs and outputs through an attention mechanism, which can break the limitation of RNN that restricts computation in parallel and learning over long distances. In addition, most of the methods are modeling temporal relations, ignoring the spatial correlation of joints, whereas a GCN can deal specifically with non-Euclidean type data, and can capture the temporal and spatial dependencies of human joints through graphs defined on temporally connected motion trees. It is understood that the transformer is not yet widely used in human motion prediction, but is well established for use in human pose estimation tasks [32, 33]. In order to use a more compact representation of a human skeleton, this study is influenced by papers [4, 27] and uses DCT coefficients for the motion transformation.

## Methods

**Fig. 1:**
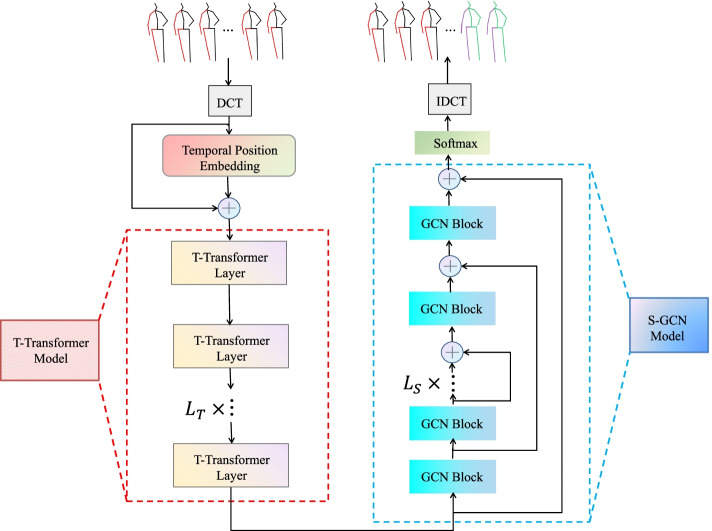
Overview of STTG-Net network structure

This study proposes a STTG-Net based on a transformer and GCN, which comprehensively considers the temporal and spatial dependence in human motion to improve the accuracy of motion prediction. The overall network framework is shown in Fig. 1.

First, the DCT is applied to encode the temporal information of each joint into the trajectory space. Second, the computed DCT coefficients are passed through a temporal position embedding (TPE) followed by a temporal transformer to learn the global dependence of the whole temporal sequence. The correlations between local joints are then efficiently learned by the spatial GCN module based on a stack of graph convolution blocks. Finally, in the testing phase, a prediction revision module is added to further correct the error of predicted action. Compared to previous models, this model captures global and local dependencies in the temporal and spatial dimensions respectively, and models the motion of human skeletal joints over time, so the prediction result is more competitive.

### Data preprocessing

Provided a motion sequence *X*_1 : __*N*_ = [*X*_1_, *X*_2_, *X*_3_, ⋯, *X*_*N*_] consisting of *N* consecutive human poses, where *X*_t_ ∈ *R*^*M*^ denotes the human pose at frame t, and *M* is the dimension size of the pose at each frame. The purpose of human motion prediction is to predict the posture sequence *X*_*N* + 1 : *N* + *T*_ for the next *T* frames. First, the last frame *X*_*N*_ is replicated *T* times to generate a temporal sequence of length *N* + *T*. In this way, the whole task becomes a matter of generating an output sequence ${\hat{X}}_{1+N+T}$ from the input sequence *X*_1 : *N* + *T*_. The DCT has the ability to obtain a more compact representation by discarding high-frequency signals, which can well capture the smoothness of human motion. Therefore, this study uses DCT to map the human motion joints into a more compact trajectory space to facilitate the learning of overall features. Let ${\left\{{X}_{k,l}\right\}}_{l=1}^L$ represent the angle data of the *k*-th joint in frames 1 to *L*, and its DCT coefficients can be calculated by the following equation:

1 $${C}_{k,l}=\sqrt{\frac{2}{L}}{\sum}_{n=1}^L{x}_{k,n}\frac{1}{\sqrt{1+{\delta}_{l1}}}\cos \left(\frac{\pi }{L}\left(n-\frac{1}{2}\right)\left(l-1\right)\right)$$

where *l* ∈ {1, 2, ⋯, *L*} , and $${\delta}_{\mathrm{ij}}=\left\{\begin{array}{c}1,i=j\\ {}0,i\ne j\end{array}\right.$$ .

Second, the computed DCT coefficients are sequentially fed into the temporal transformer (T-transformer) and spatial GCN (S-GCN) modules to learn the dependencies in the temporal and spatial dimensions respectively. Finally, the processed DCT coefficients are subjected to an inverse discrete cosine transform (IDCT) to obtain the human motion pose data, with the following equation:

2 $${\mathrm{x}}_{k,n}=\sqrt{\frac{2}{L}}{\sum}_{l=1}^L{C}_{k,L}\frac{1}{\sqrt{1+{\delta}_{l1}}}\cos \left(\frac{\pi }{L}\left(n-\frac{1}{2}\right)\left(l-1\right)\right)$$

### T-transformer

Compared with an RNN commonly used in human motion prediction, the transformer has a relatively improved ability to extract long-distance features and can build long dependencies dynamically on input sequences. Therefore, it can more effectively capture the long-distance dependencies. Considering these advantages, the use of a transformer is proposed instead of an RNN and other variant networks used in the past to capture the relationship between more frames in the temporal dimension in order to obtain more temporal dependence. Unlike [34] using a spatio-temporal transformer, this study builds a network based on transformers only in the temporal dimension, therefore, the temporal transformer (T-transformer) module is proposed.

#### T-transformer module

**Fig. 2:**
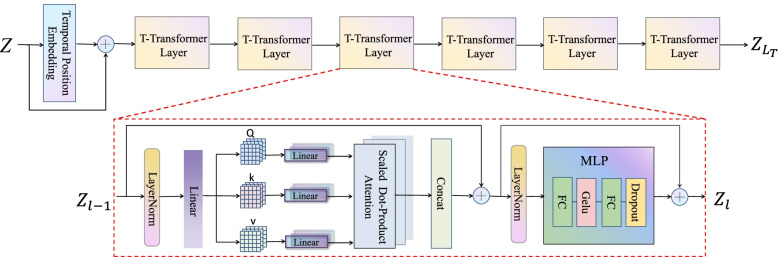
Temporal transformer (T-transformer) module. The module combines the encoded features of the connected human pose vector Z through the TPE and the input sequence, and obtains the output ${\mathrm{Z}}_{{\mathrm{L}}_{\mathrm{T}}}$ through the T-transformer module composed of 6 identical T-transformer layers. Specifically, each T-transformer layer will through the layer norm, and then the multi-head attention calculation is performed by the dot product attention composed of Q, K, and V of multiple heads, and finally connect the attention results and pass through the MLP composed of two FC layers

The proposed T-transformer module focuses on modeling the global dependencies between temporal frames in the input sequence and the network structure, as is shown in Fig. 2. Similar to the machine translation task, when using the transformer, the human pose is regarded as a ‘word’ and then the future pose is predicted in the same way as the ‘word.’ The sequence of human poses {*X*_1_, *X*_2_, ⋯, *X*_*N* + *T*_} is concatenated with *Z* ∈ *R*^(*N* + *T*) × *K*^ after the DCT, where *K* is the dimension of each pose. Before the T-transformer module is applied, in order to retain the position information of the temporal frames, the TPE is used, and then the result is added to the input sequence to obtain the input feature *Z*_0_ ∈ *R*^(*N* + *T*) × *K*^. The T-transformer encoder consists of a multi-headed dot product attention and multilayer perceptron (MLP) to focus on the temporal correlation of the input data, and its output is denoted as ${Z}_{L_T}\in {R}^{\left(N+T\right)\times K}$. The whole temporal transformer structure can be expressed as the following process:

3 $${Z}_0= TPE(Z)+Z$$

4 $${Z}_{\mathrm{ma}}= MA\left( LN\left({Z}_{l-1}\right)\right)+{Z}_{l-1}$$

5 $${Z}_{\mathrm{l}}= MLP\left( LN\left({Z}_{ma}\right)\right)+{Z}_{ma}$$

where *LN*(⋅) represents layer normalization, and *l* = 1, 2, ⋯, *L*_*T*_ denotes that the T-transformer is stacked by *L*_*T*_ equal layers.

#### Multi-head self-attention

The use of multi-head attention is intended to simulate information from subspace with different locations using multiple heads. The input feature *Z*_0_ ∈ *R*^(*N* + T) × *K*^ will be calculated by a linear transformation to obtain *Q* = *ZW*_*Q*_, *K* = *ZW*_*K*_, and *V* = *ZW*_*V*_ , where the weight matrixs *W*_*Q*_, *W*_*K*_, *W*_*V*_ ∈ *R*^*K* × *K*^, and *Q*, *K*, *V* ∈ *R*^(*N* + *T*) × *K*^. Then the three input matrices *Q*, *K*, *V* are subjected to h different linear transformations (h represents the number of used heads), and the dot product attention is used for parallel processing. Finally, the attention outputs of the h heads are concatenated together. This process can be expressed as:

6 $${H}_i= Attention\left({Q}_{\mathrm{i}},{K}_i,{V}_i\right),i\in \left[1,\cdots, h\right]$$

7 $$MA\left(Q,K,V\right)=\mathrm{concat}\left({H}_1,{H}_2,\cdots, {H}_h\right){W}_{out}$$

where *W*_*out*_ is the weight matrix of the attention output of the spliced h heads and h indicates the number of multiple heads, in this study h takes the value of 8.

#### Scaled dot-product attention

The dot-product attention model used in this study is the scaled dot product attention [31]. This attention can be interpreted as an input composed of query matrix *Q*, key matrix *K*, and value matrix *V*. The attention output is computed by calculating the dot product of each query and all keys, its dot product result is multiplied by a certain scaling factor, and then the weight of value is obtained by the Softmax function. The similarity score between *Q* and *K* can be calculated as follows:

8 $$S\mathrm{co} re=\frac{QK^T}{\sqrt{d}}$$

where $1 \left/ \sqrt{d}\right.$ is the scaling factor. The aim is to perform proper normalization to prevent the value of d from increasing, which will cause the use of the Softmax function to saturate and only produce a very small gradient. Ultimately, the output obtained after the dot product attention can be expressed as:

9 $$Attention\left(Q,K,V\right)=\mathrm{Softmax}\left(\frac{QK^T}{\sqrt{d}}\right)V$$

#### MLP

The MLP is added to increase the non-linearity of the network. In this study, the output of multi-head attention is used as the input of the MLP after layer normalization and then passed through two fully connected layers in turn, which can be expressed as follows:

10 $$MLP\left( LN\left({Z}_{\mathrm{ma}}\right)\right)=\mathrm{dropout}\left( fc\left( gelu\left( fc\left( LN\left({Z}_{ma}\right)\right)\right)\right)\right)$$

where *LN*(⋅) denotes layer normalization, fc(⋅) denotes a fully connected layer, and *Z*_*ma*_ is the output of a multi-headed self-attentive layer.

### S-GCN

**Fig. 3:**
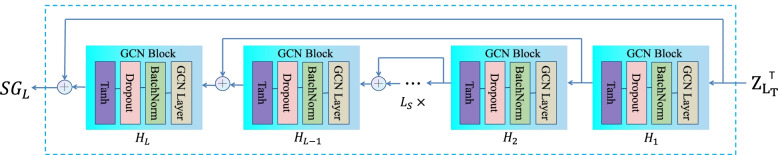
The architecture of S-GCN module

The proposed T-transformer module can only extract the temporal features of the sequence. However, because of the motion coupling, joints also affect each other in space during motion. Considering that the human skeleton is similar to the graph structure in the data structure, its joints can be regarded as nodes of the graph and the connections between joints can be considered as edges. Inspired by ref. [35], this study adopts the GCN module which is similar to refs. [4, 27]. The network structure is improved as shown in Fig. 3, namely, S-GCN. The human skeleton is regarded as a fully connected graph with *K* nodes, the learnable adjacency matrix *A* ∈ *R*^*K* × *K*^ represents the connection strength between the nodes, the feature matrix *H*^(*l*)^ ∈ *R*^*K* × *K*^ is the input of the graph convolution layer, M represents feature dimension of the output of the previous layer. In addition, the output of the graph convolution block can be obtained by combining the trainable weights $\tilde{M}$ is the feature dimension of the output of the graph convolution layer, and the entire graph convolution block can be expressed as follows:

11 $${H}^{l+1}=\tanh \left( dropout\left( BN\left({A}^{\left(\mathrm{l}\right)}{H}^{(l)}{W}^{(l)}\right)\right)\right)$$

where *BN*(⋅) means batch normalization. Either *A*^(*l*)^ or *W*^(l)^ can be obtained by back propagation training.

The *K* × (*N* + *T*) matrix of output by the T-transformer was used as the first layer input of S-GCN, and after each graph convolution block, a $K\times \tilde{M}$ size matrix would be obtained. The S-GCN module was constructed by designing to stack multiple such graph convolution blocks. To match the dimension size, the dimension of the last layer was mapped back to the same dimension as the input matrix, and the output of the whole S-GCN module was denoted as ${SG}_{L_S}\in {R}^{K\times \left(N+T\right)}$. Adding long residual connections [36] between the *i*-th and (*L*-*i* + 1)-th block was considered, *i* ∈ (1, ⋯, *L*/2), as shown in Fig. 3. Adding long residual connections allows for easier propagation of gradients, prevents gradient disappearing, and accelerates training.

### Prediction revision module

**Fig. 4:**
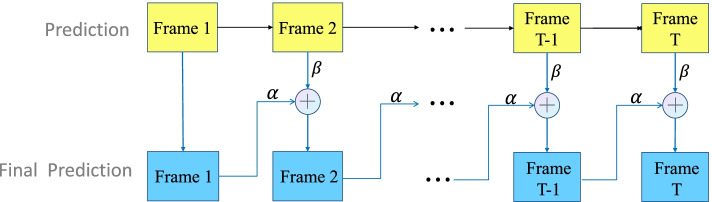
The prediction revision module. The first line of ‘prediction’ is the result directly predicted by the network, while the second line is the “final prediction.” From the second frame, the current ‘prediction’ result is fused with the “final prediction” result of the previous frames and then calculated as the final prediction for the current frame

A common problem in human motion prediction is that it is difficult to recover from its predicting error, which leads to error accumulation and discontinuous motions. Previous works have commonly addressed this problem by sampling-based loss [14] and convergence loss [21], or by forcing the internal state of the network through a GAN, both of which increase the hyper-parameter of the network to a certain extent. Unlike previous works, this study adds a simple and effective prediction revision module in the testing phase to reduce the final prediction error of the model, as shown in Fig. 4. The module is based on a fusion strategy, in which the current prediction frame is fused with the prediction information from the previous frame, and then the fused value is used as the prediction value for the current frame. The basis for this consideration is that human actions are continuous, and the difference in actions between two adjacent frames should not be too great. So if the current frame produces a large prediction error, fusion with the prediction of the previous frame will ‘pull’ back the prediction of the current frame to prevent a sudden jump in motion. Thereby the prediction error is reduced and the smoothness of motion is improved. The specific fusion equation is shown below:

12 $$\hat{Y}=\alpha {\hat{Y}}_P+\beta {\hat{Y}}_C$$

where ${\hat{Y}}_P$ is the predicted value of the previous frame, ${\hat{Y}}_C$ is the ‘predicted’ value of the current frame, $\hat{Y}$ represents the ‘final predicted’ value of the current frame, and *α* and *β* are fusion coefficients.

## Results and Discussion

In order to demonstrate the effectiveness of STTG-Net proposed in this study, experiments were carried out on the Human3.6 M dataset. The results were compared and analyzed with the state-of-the-art method.

### Experimental details

The proposed network model was implemented based on Pytorch framework and trained it using the ADAM optimizer [37]. All experimental results were obtained by using a single NVIDIA 1080Ti graphics card. The batch size was set to 32, the number of training epochs was set to 3000 and the learning rate was 0.0005. The parameter size of the network was 2.33 M.

Joint angles were used to represent the human pose. Given the input joint angles, the corresponding coefficients were obtained by using DCT and then applying IDCT to recover the predicted DCT coefficient to the corresponding angle after training the model. In order to train the network effectively, the average *L*_1_ distance between the predicted joint angle and the ground truth was applied as the loss function. Thus, for a training sample, the loss function can be expressed as:

13 $${L}_1=\frac{1}{\left(N+T\right)K}{\sum}_{\mathrm{n}=1}^{N+T}{\sum}_{k=1}^K\left|{\hat{x}}_{k,n}-{x}_{k,n}\right|$$

where ${\hat{\mathrm{x}}}_{k,n}$ is the predicted value of the *k*-th joint in the *n*-th frame obtained through the network, and x_*k*, *n*_ is its corresponding ground truth.

### Dataset

Human3.6 M [38] is currently the most commonly used open-source dataset in human motion prediction task. It contains 3.6 million 3D human pose data recorded by the Vicon motion capture system and the corresponding RGB images, depth images, and body surface data acquired by 3D scanning equipment. It describes 15 actions such as walking, eating, discussing, and more, which are performed by seven subjects, each subject performs two experiments for each action, with each a sequence containing approximately 3000 to 5000 frames, and each frame contains 34 rows of data, including global translation, global rotation and 32 joint rotations relative to their parent joints. According to the data processing of previous works [4, 30], global rotation, translation, and constant angle were removed. Following standard agreements [13, 14, 26], all motion sequences were down sampled to 25 frames per second, Subject 5(S5) was used as a test set, Subject 11(S11) was used as validation set, and the remaining subjects were used as a training set.

### Evaluation metric and baselines

#### Evaluation metric

In order to fairly verify the validity of experimental results, mean angular error (MAE) was used as the evaluation metric. Specifically:

14 $$MAE=\frac{1}{N}{\sum}_{i=1}^N\left|{\hat{y}}_n-{y}_n\right|$$

where ${\hat{\mathrm{y}}}_n$ is the predicted value of n-th frame, and y_*n*_ is its corresponding ground truth. For the above evaluation metric, the prediction results from 0 to 400 ms were highlighted and reported following the baselines of previous works [13, 14].

#### Baselines

The proposed approach was compared with commonly used motion prediction baselines and some of the latest methods, including Multi-Gan [39], OoD [40], ST-Conv [29], POTR-GCN [30] and ST-transformer [34] as well as the state-of-the-art methods HRI [27] and DMGNN [22]. For the used prediction baselines, the results were taken from their respective papers, and for HRI [27], the official code published on GitHub was reproduced.

### Experimental results

Consistent with previous studies, the model was trained using 50 frames and predicted the pose for the next 10 frames. Table 1 [3, 14, 22, 27, 29, 30, 34, 39, 40] shows the joint angle error results of all actions compared with baselines of this model on Human3.6 M. In order to observe the results more intuitively, the best results among all the experimental results are presented in bold, and the sub-optimal results are presented in italics.

**Table 1:** The joint angle error and average angle error of all actions compared with baselines on Human3.6M

Milliseconds	80	160	320	400	80	160	320	400	80	160	320	400	80	160	320	400
	Walking				Eating				Smoking				Discussion			
Res. sup. [14]	0.28	0.49	0.72	0.81	0.23	0.39	0.62	0.76	0.23	**0.39**	**0.62**	**0.76**	0.31	0.68	1.01	1.09
convSeq2Seq [3]	0.33	0.54	0.68	0.73	0.22	0.36	0.58	0.71	0.26	0.49	0.96	0.92	0.32	0.67	0.94	1.01
Multi-Gan [39]	0.23	0.51	0.62	0.66	0.20	0.31	**0.49**	0.66	0.25	0.46	0.88	0.88	0.28	0.55	0.81	0.92
OoD [40]	0.23	0.37	0.58	0.63	0.21	0.37	0.59	0.72	0.27	0.54	1.03	1.03	0.30	0.66	0.94	1.02
DMGNN [22]	*0.18*	*0.31*	*0.49*	0.58	0.17	*0.30*	**0.49**	**0.59**	*0.21*	**0.39**	*0.81*	*0.77*	0.26	0.65	0.92	0.99
ST-Conv [29]	0.19	0.34	0.57	0.63	*0.16*	**0.29**	*0.50*	*0.60*	0.22	0.41	0.85	0.81	0.22	0.57	0.84	0.98
POTR-GCN [30]	**0.16**	0.40	0.62	0.73	**0.11**	**0.29**	0.53	0.68	**0.14**	**0.39**	0.84	0.82	**0.17**	*0.52*	0.79	0.88
ST-Transformer [34]	0.21	0.36	0.58	0.63	0.17	*0.30*	**0.49**	*0.60*	0.22	0.43	0.88	0.82	*0.19*	*0.52*	0.79	0.88
HRI [27]	*0.18*	**0.30**	**0.46**	**0.51**	*0.16*	**0.29**	**0.49**	*0.60*	0.22	*0.40*	0.86	0.80	0.20	*0.52*	*0.78*	*0.87*
Ours	0.19	0.33	*0.49*	*0.57*	*0.16*	*0.30*	0.51	0.62	0.33	0.61	1.05	1.15	0.21	**0.47**	**0.71**	**0.78**
	Direction				Greeting				Phoning				Posing			
Res. sup. [14]	0.26	0.47	0.72	0.84	0.75	1.17	1.74	1.83	**0.23**	**0.43**	**0.69**	**0.82**	0.36	0.71	1.22	1.48
convSeq2Seq [3]	0.39	0.60	0.80	0.91	0.51	0.82	1.21	1.38	0.59	1.13	1.51	1.65	0.29	0.60	1.12	1.37
Multi-Gan [39]	0.36	0.57	-	0.89	0.51	0.86	-	1.36	0.54	1.05	-	1.58	0.22	0.51	-	1.41
OoD [40]	0.38	0.58	0.79	0.90	0.49	0.81	1.24	1.43	0.57	1.10	1.48	1.61	0.26	0.56	1.26	1.55
DMGNN [22]	0.32	0.65	0.93	1.05	0.36	0.61	*0.94*	*1.12*	0.52	0.97	1.29	1.43	*0.20*	**0.46**	1.06	1.34
ST-Conv [29]	*0.24*	*0.43*	0.77	0.81	0.35	0.61	1.01	1.20	0.53	1.00	*1.28*	1.40	0.26	0.51	1.08	1.32
POTR-GCN [30]	**0.20**	0.45	0.79	0.91	**0.29**	0.69	1.17	1.30	0.50	1.04	1.41	1.54	0.61	0.68	1.05	*1.28*
ST-Transformer [34]	0.25	**0.38**	0.75	0.86	0.35	0.61	1.10	1.32	0.53	1.04	1.41	1.54	0.61	0.68	*1.05*	*1.28*
HRI [27]	0.25	*0.43*	**0.60**	**0.69**	0.35	*0.60*	0.95	1.14	0.53	1.01	1.31	1.43	**0.19**	**0.46**	1.09	1.35
Ours	0.27	*0.43*	*0.67*	*0.76*	*0.33*	**0.59**	**0.90**	**1.05**	*0.42*	*0.82*	1.29	*1.16*	0.25	*0.49*	**1.02**	**1.24**
	Purchases				Sitting				Sittingdown				Takingphoto			
Res. sup. [14]	0.51	0.97	1.07	1.16	0.41	1.05	1.49	1.63	0.39	0.81	1.40	1.62	0.24	0.51	0.90	1.05
convSeq2Seq [3]	0.63	0.91	1.19	1.29	0.39	0.61	1.02	1.18	0.41	0.78	1.16	1.31	0.23	0.49	0.88	1.06
Multi-Gan [39]	0.55	0.85	-	1.23	0.35	0.60	-	1.13	0.36	0.72	-	1.20	0.23	0.41	-	0.99
OoD [40]	0.61	0.89	1.27	1.37	0.38	0.62	1.06	1.22	0.41	0.83	1.28	1.41	0.25	0.51	0.81	0.95
DMGNN [22]	**0.41**	**0.61**	1.05	1.14	**0.26**	**0.42**	**0.76**	*0.97*	0.32	0.65	0.93	1.05	*0.15*	**0.34**	*0.58*	0.71
ST-Conv [29]	*0.42*	**0.61**	1.08	1.15	0.30	0.49	0.90	1.09	*0.29*	0.65	0.97	1.08	*0.15*	**0.34**	*0.58*	0.72
POTR-GCN [30]	0.33	0.63	1.04	1.09	*0.25*	0.47	0.92	1.09	**0.25**	*0.63*	1.00	1.12	**0.12**	0.41	0.71	0.86
ST-Transformer [34]	0.43	0.77	1.30	1.37	0.29	0.46	0.84	1.01	0.32	0.66	0.98	1.10	*0.15*	0.38	0.64	0.75
HRI [27]	*0.42*	0.65	*1.00*	*1.07*	0.29	0.47	0.83	1.01	0.30	*0.63*	*0.92*	*1.04*	0.16	0.36	*0.58*	*0.70*
Ours	0.43	*0.62*	**0.90**	**0.96**	0.30	*0.45*	*0.77*	**0.94**	0.40	**0.45**	**0.77**	**0.94**	0.16	*0.35*	**0.57**	**0.69**
	Waiting				Walkingdog				Walkingtogether				Average			
Res. sup. [14]	0.28	0.53	1.02	1.14	0.56	0.91	1.26	1.40	0.31	0.58	0.87	0.91	0.36	0.67	1.02	1.15
convSeq2Seq [3]	0.30	0.62	1.09	1.30	0.59	1.00	1.32	1.44	0.27	0.52	0.71	0.74	0.38	0.68	1.01	1.13
Multi-Gan [39]	0.23	0.56	-	1.29	0.53	0.85	-	1.33	0.22	0.45	-	0.73	0.37	0.67	-	1.43
OoD [40]	0.29	0.58	1.06	1.29	0.52	0.88	1.17	1.34	0.21	0.44	0.66	0.74	0.37	0.63	1.08	1.18
DMGNN [22]	0.22	*0.49*	*0.88*	*1.10*	*0.42*	**0.72**	1.16	1.34	*0.15*	*0.33*	**0.50**	0.57	*0.27*	*0.52*	0.83	0.95
ST-Conv [29]	*0.21*	0.51	0.97	1.17	0.43	0.78	1.10	1.24	*0.15*	**0.32**	**0.50**	**0.54**	*0.27*	*0.52*	0.87	0.98
POTR-GCN [30]	**0.17**	0.56	1.14	1.37	**0.35**	0.79	1.21	1.33	*0.15*	0.44	0.63	0.70	**0.22**	0.56	0.94	1.01
ST-Transformer [34]	0.22	0.51	0.98	1.22	0.43	0.78	1.15	1.30	0.17	0.37	0.58	0.62	0.30	0.55	0.90	1.02
HRI [27]	0.22	*0.49*	0.92	1.14	0.46	0.78	*1.05*	*1.23*	**0.14**	**0.32**	**0.50**	*0.55*	*0.27*	*0.52*	*0.82*	*0.94*
Ours	0.24	**0.47**	**0.85**	**1.04**	0.43	*0.73*	**1.03**	**1.18**	0.17	0.36	*0.51*	0.57	0.28	**0.50**	**0.80**	**0.89**

The best results are presented in bold, and the sub-optimal results are presented in italics

It can be seen from the comparison results that compared with the common baselines [3, 14] in motion prediction, STTG-Net has made great improvements in all other actions except for the ‘Phoning’ movement. This is mainly due to the fact that the ‘Phoning’ movement has less spatio-temporal dependence, as its movements occur mainly at one hand and the rest of the body is almost static. Even so, STTG-Net achieved sub-optimal results on this action. Compared with the recently proposed method [34] that also uses transformers for motion prediction, the error produced by the proposed method is almost smaller for each action, resulting in better average error results. Because ST-transformer [34] pays more attention to long-term prediction, it performs better in motions longer than 1 s, indicating that the advantage of the spatio-temporal transformer is more obvious as time increases. The study focused more on short-term motion prediction and only used temporal transformer to capture the temporal relationship, producing excellent results in short-term forecasting, which shows the effectiveness of the temporal transformer in this study. For other recently proposed methods proposed in refs. [22, 29, 30, 34, 39, 40], STTG-Net achieved optimal results on more than half of the actions and achieved approximate optimal results on the others. In the comparison of the average error, in addition to the sub-optimal results at 80 ms, STTG-Net achieved the best results at 160, 320, and 400 ms, respectively. Moreover, compared with the state-of-the-art method, the average prediction error was reduced by 3.85% at 160 ms, 2.44% at 320 ms, and 5.32% at 400 ms. Furthermore, it can be seen from the experimental results that the prediction error of STTG-Net grew slower with the prediction time increase, which indicates that the method has a small error accumulation.

**Fig. 5:**
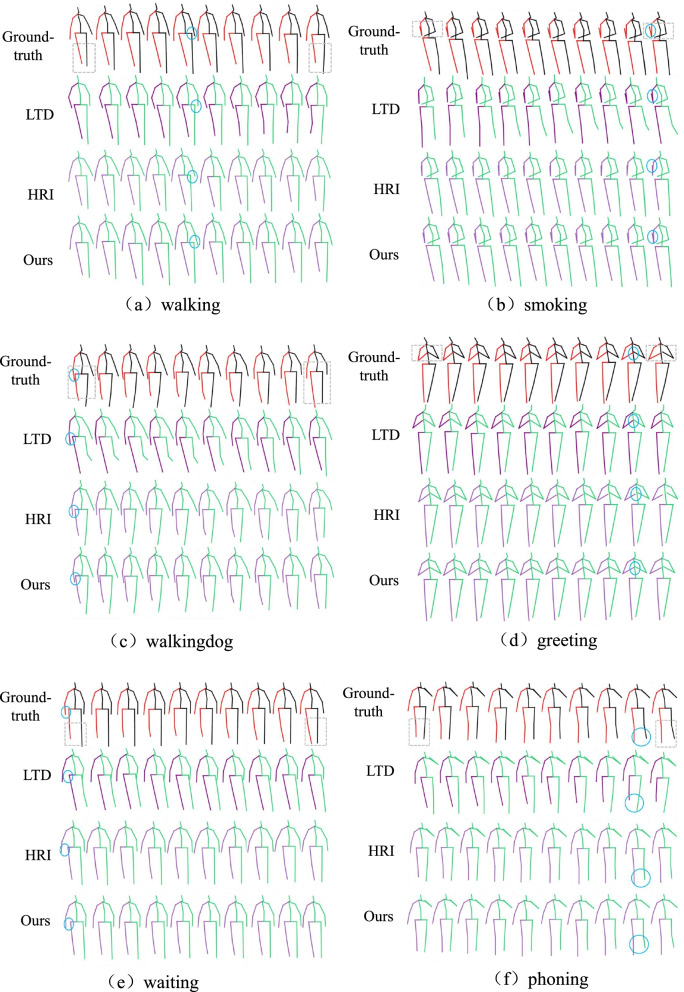
Visualization results of predictions for the four actions of (a) walking (b) smoking (c) walkingdog (d) greeting (e) eating (f) phoning on Human3.6 M. The ground-truth, LTD [4], HRI [27], and the proposed method are shown from top to bottom. The changes in actions from the first to the last frame of the prediction can be clearly seen in the grey dashed box, while the blue round box shows the comparison between predicted action and ground truth by the proposed method and other methods. It can be seen from the visualization results that the proposed method produces predictions closer to the ground truth than HRI and LTD.

Since STTG-Net adopted the transformer structure, the model is relatively simple and has fewer parameters. The total parameter amount is only 2.33 M, whereas the total parameter amount of ref. [27] is 3.08 M. In order to more intuitively show the advantages of the method in this study, a visual comparison of some prediction results was made, and the comparison results are shown in Fig. 5.

### Ablation experiment

This study conducted extensive ablation experiments on the Human3.6 M dataset to better validate the contributions of various module components in the proposed STTG-Net. In order to compare the impact of the validation component more fairly, the structural parameters were fixed, except for the part to be verified in the experiment.

#### The influence of the T-transformer module, S-GCN module, and prediction revision module

In Method section, the proposed T-transformer module, S-GCN module, and prediction revision module was described in detail, and here the focus was on evaluating their impact on the whole network. The DCT coefficients were used as the input of T-transformer module, and a TPE was also included before the input module, with the aim of retaining more information about the position of temporal frames, so the impact of TPE was evaluated simultaneously. To prove the effects of each proposed modules, the following combinations of ablation experiments were explored: (1) applying only the T-transformer module; (2) applying only the S-GCN module; (3) using the T-transformer and S-GCN module; (4) using T-transformer, S-GCN, and TPE; (5) using T-transformer, S-GCN, TPE and the prediction revision module (PR for short). The results are documented in Table 2.

**Table 2:** The ablation studies of different modules in STTG-Net, reporting results for the joint angle errors on Human3.6M. “√” indicates that the module is used in experiment, “×” means that the part is removed from experiment

	T-Transformer	S-GCN	TPE	PR	MAE			
					80 ms	160 ms	320 ms	400 ms
(1)	√	×	×	×	0.59	0.83	1.16	1.28
(2)	×	√	×	×	0.49	0.69	0.98	1.09
(3)	√	√	×	×	0.28	0.54	0.88	1.00
(4)	√	√	√	×	**0.27**	0.52	0.86	0.98
(5)	√	√	√	√	0.28	**0.50**	**0.80**	**0.89**

The best results are presented in bold

Based on the results of the ablation experiment, it could be seen that when T-transformer and S-GCN were used together, the effect was better than using either one alone. It further proved that T-transformer and S-GCN could capture the dependencies in temporal and spatial dimensions respectively. When the TPE module was used, the prediction results reached the level of state-of-the-art. After adding the prediction revision module, the prediction error at 80 ms increased by 0.01. This is mainly since for prediction results with small error, the revision module may introduce new error. However, for the other cases, the prediction results were improved to a different extent. Especially when the error is large, the revision effect is obvious.

#### The influence of network parameters

There are three important parameters in the STTG-Net. They are the number of multi-heads H, the layer number of T-transformer L_T_, and the layer number of G-GCN L_S_. The experiment also explored various combinations of these parameters to find the best composition of the network structure. Without adding the prediction revision module, the effect of different structural parameters on experimental results is recorded in Table 3. From the results, it is easy to find that, when H = 8, L_T_ = 6, and L_S_ = 14, the network acquired the best result.

**Table 3:** The ablation experiments for different module parameters in STTG-Net, with reported results for joint angle errors on Human3.6M

H	8	6	12	8	8	8	8
L_T_	6	6	6	4	8	6	6
L_S_	14	14	14	14	14	12	16
80 ms	**0.27**	0.28	0.28	0.28	0.28	0.29	0.27
160 ms	**0.52**	0.55	0.53	0.53	0.55	0.54	0.53
320 ms	**0.86**	0.87	0.86	0.86	0.89	0.87	0.87
400 ms	**0.98**	0.99	0.99	0.98	1.01	1.00	0.99

The best results are presented in bold

#### Influence of coefficients in prediction revision module

During the experiment, the fusion effect of the predicted frame and the predicted value of the previous frame were investigated, so different combinations of α and β coefficients were used to explore the optimal degree of fusion. Table 4 shows the results of different coefficients. Through experiments, it was found that different experimental coefficients had different effects on the prediction results. When α = 0.125 and β = 0.875, the smallest mean error is obtained. Therefore, this group of coefficients was selected in the final experiment.

**Table 4:** The ablation studies with different coefficients in prediction revision module, reporting results for the mean joint angle errors at 80, 160, 320, and 400 ms for different α and β on Human3.6M

	80 ms	160 ms	320 ms	400 ms
α=0, β=1	**0.27**	0.52	0.86	0.98
α=0.1, β=0.9	0.30	0.54	0.88	0.99
α=0.125, β=0.875	0.28	**0.50**	**0.80**	**0.89**
α=0.175, β=0.825	0.30	0.54	0.85	0.97
α=0.225, β=0.775	0.29	0.54	0.86	0.98
α=0.25, β=0.75	0.28	0.53	0.86	0.99
α=0.5, β=0.5	0.29	0.55	0.88	1.00

The best results are presented in bold

## Conclusions

The spatio-temporal network (STTG-Net) proposed in this work used its internal T-transformer and S-GCN two modules to model the spatio-temporal dependence of human skeletal joints, and the prediction revision module can reduce the cumulative error by fusing the current prediction frame with the prediction information of the previous frame to better accomplish the task of human motion prediction. The experiments on the Human3.6 M dataset show that the proposed method achieved state-of-the-art results on most actions compared to the commonly used baselines and recently released motion prediction models. Although STTG-Net produced excellent results in short-term motion prediction using relatively few parameters, there remains still room to reduce the amount of parameters and improve the results in long-term motion prediction. For future work, we will continue to try to build a lightweight network to further reduce network parameters, and study algorithms to learn the fusion changes of correction modules. Further we will continue to explore models for longer-term motion prediction.

## Supplementary Information

**Additional file 1.**

## Data Availability

The datasets used or analyzed during current study are public available.

## Abbreviations

STTG-Net: Spatio-temporal network based on transformer and GCN
GCN: Graph convolutional network
GAN: Generative adversarial network
RNN: Recurrent neural network
CNN: Convolutional neural network
LSTM: Long short-term memory
DCT: Discrete cosine transform
TPE: Temporal position embedding
ICDT: Inverse discrete cosine transform
T-transformer: Temporal transformer
MLP: Multilayer perceptron
MAE: Mean anqular error
GRU: Gated recurrent unit
S-GCN: Spatial GCN
